# Norethisterone-Induced Liver Injury and a Short Survey Among Gynecologists

**DOI:** 10.7759/cureus.40300

**Published:** 2023-06-12

**Authors:** Jawad Iqbal Rather, Muzafar Maqsood Wani, Khalid Bin Mushtaq Lone, Rabiya Rasheed

**Affiliations:** 1 Nephrology, Sher-i-Kashmir Institute of Medical Sciences, Srinagar, IND; 2 Internal Medicine, Sher-i-Kashmir Institute of Medical Sciences, Srinagar, IND; 3 Pathology, Government Medical College, Srinagar, IND

**Keywords:** norethisterone induced liver injury, cholestasis, menorrhagia, norethisterone, drug-induced liver injury (dili)

## Abstract

Norethisterone, a commonly used oral contraceptive, and treatment for various gynecological disorders such as menorrhagia, abnormal uterine bleeding, and breast cancer, has been associated with multiple liver injuries. These injuries can manifest as hepatitis or cholestatic types of injury, benign neoplasms, peliosis hepatis, sinusoidal obstruction syndrome, and enlargement of existing hemangiomas. This report presents three cases in which liver enzyme levels were elevated due to norethisterone intake. Two of the cases were individuals undergoing evaluation as potential kidney donors in the nephrology department for their spouses, while the third case involved a patient with chronic kidney disease (CKD) stage-5 on maintenance hemodialysis. Regular follow-up of these patients, particularly due to the significance of two being kidney donors and one having advanced CKD, allowed for early detection of asymptomatic liver enzyme elevation and prompt discontinuation of norethisterone. Prescribing norethisterone is common in gynecological settings, including ours. To assess gynecologists' knowledge regarding norethisterone-related side effects, we conducted an online survey, the results of which are discussed in this report.

## Introduction

Norethisterone, a commonly prescribed progesterone has been associated with a spectrum of liver injury including hepatitis, cholestasis, benign neoplasia, peliosis, hepatic veno-occlusive disease, and enlargement of hemangiomas. We report norethisterone-induced hepatitis in three women (two potential kidney donors and a chronic kidney disease patient on maintenance hemodialysis), which resolved promptly on discontinuation of norethisterone. An online survey revealed that many gynecologists who frequently prescribe norethisterone were unaware of the potential hepatotoxicity of norethisterone. Periodic liver function test monitoring of patients on norethisterone is suggested.

## Case presentation

Case 1

A 40-year-old woman with no known comorbidity was admitted to the kidney transplant unit as a potential kidney transplant donor and had documented normal routine investigations including liver function tests (LFT). Two weeks prior to her admission, a gynecologist had started her on norethisterone 5 mg daily for menorrhagia. The history and clinical examination at the time of admission were unremarkable. She did not consume alcohol or any other drug or herbal products. Investigations revealed elevated liver enzymes as shown in Table 1. Coagulogram was normal. Serologies for hepatitis A, B, C, & E, cytomegalovirus (CMV), and Epstein-Barr virus (EBV) were non-reactive. Screening for Wilson’s disease, hemochromatosis, and autoimmune hepatitis were negative. Ultrasound of the abdomen showed a normal-sized liver with normal echotexture: the rest of the abdominal and pelvic viscera, including the uterus and adnexa, were normal. Significantly her baseline LFT done prior as part of her donor workup was normal with alanine transaminase (ALT) of 29 units/L. Her ALT peaked at 862 units/L after which it started decreasing and normalized over a period of around two weeks. 

Case 2

A 35-year-old woman with no known comorbidity was admitted as a voluntary kidney transplant donor. The patient had a history of menorrhagia one month before admission for which she sought local physician consultation and was started on norethisterone 5mg daily. There was no history of urine discoloration, icterus, or consumption of any drug, alcohol, or herbal products. Her physical examination was normal. Laboratory parameters are depicted in Table 1, which show a high ALT and aspartate transaminase (AST). Her LFT prior to the initiation of norethisterone was normal. Coagulogram was normal. Serologies for hepatitis A, B, C, & E, CMV, and EBV were non-reactive. Screening for Wilson’s disease, hemochromatosis, and autoimmune hepatitis was negative. Ultrasound of the abdomen showed a normal hepatobiliary system, kidneys, and other pelvic organs. Norethisterone was stopped and her LFTs were serially monitored which resolved after the drug was stopped.

Case 3

A 28-year-old woman with chronic kidney disease on maintenance hemodialysis was admitted for a kidney transplant. Routine investigations revealed anemia and transaminitis (Table 1). She was screened for hepatitis B & C with HBV DNA and HCV RNA respectively which were negative. On reviewing her records, she was found to have taken norethisterone prescribed by a gynecologist 10 days prior. Laboratory parameters at the presentation are mentioned in Table 1. As part of her routine monthly test in the dialysis unit, her LFT a month before was normal. The coagulation profile was normal. Serologies for hepatitis A, B, C, & E, CMV, and EBV were non-reactive. Screening for hemochromatosis was negative. Her AST and ALT normalized within two weeks of stopping norethisterone.

**Table 1:** Labs at the time of presentation in cases 1, 2, and 3 ALT: alanine transaminase, AST: aspartate transaminase, ALP: alkaline phosphatase

Test	Case 1	Case 2	Case 3
Hemoglobin (g/dL)	11.7	12.7	8.7
WBC	4.1	4.5	7.5
Platelet (×10^9^)	119	138	210
Glucose (mg/dL)	84	97	88
Urea	16	33	123
Creatinine	0.85	0.98	6.98
Bilirubin	0.55	0.45	0.95
ALT	424	154	111
AST	223	98	99
ALP	92	78	178
Albumin	4.14	4.14	3.5

## Discussion

Norethisterone is a progesterone hormone that finds frequent usage in contraception and the management of specific gynecological conditions. These conditions may include menorrhagia, the postponement of menstruation, abnormal uterine bleeding, and breast cancer treatment [1]. Female sex hormones or oral contraceptives are known to cause several types of liver injuries including hepatitis or cholestasis, benign neoplasms, etc [2].

Norethisterone has both estrogenic and androgenic effects [3,4]. There are reports of hepatitic-type injury with androgens, so some of the hepatotoxicity caused by norethisterone can be explained by its androgenic properties [5]. Another mechanism, as demonstrated by animal studies, suggests that progesterone has the potential to elevate the production of proinflammatory cytokines. These cytokines might play a role in the development of progesterone-induced hepatic injury [6].

In a case series involving three patients with norethisterone-related hepatitis, elevated levels of ALT and AST were observed, surpassing ten times the upper limit of normal. However, within two weeks of discontinuing the drug, these levels gradually returned to the near-normal range, aligning with the trends observed in our cases [7].

In the available literature, several case reports have documented the hepatic effects of norethisterone, including instances of cholestasis [8] or jaundice [9]. However, there are only a limited number of reports discussing isolated cases of elevated liver transaminases as a result of norethisterone use.

In our three cases, norethisterone intake for menorrhagia was the only significant history. The history, physical examination, and laboratory findings excluded any other possible etiology. The temporal association of norethisterone intake and abnormality in liver enzymes with the resolution of the abnormality on drug discontinuation strongly favors norethisterone-induced liver injury in our patients. There was no other predisposing factor like non-alcoholic fatty liver disease or alcohol use. Previous case reports suggest an increased risk of norethisterone-induced liver injury in patients with underlying predisposing factors [10,11].

The Roussel Uclaf Causality Assessment Method (RUCAM) was utilized in our case to assess the causality of drug-induced liver injury. RUCAM is a standardized tool used for suspected cases of drug-induced liver injury and evaluates various factors such as the timing of liver injury in relation to drug initiation, concomitant use of medications with potential hepatotoxicity, exclusion of non-drug-related causes of liver injury, response to drug withdrawal, response to re-challenge with the drug, and specific risk factors (such as alcohol consumption, age, and pregnancy). Each case is assigned a score ranging from -9 to +14, with a score of ≤3 indicating an unlikely association, 4-5 suggesting a possible association, 6-8 indicating a probable association, and >8 indicating a highly probable association with drug-induced hepatotoxicity [12]. In our cases, the RUCAM scores were +8 for cases 1 and 2, and +7 for case 3, indicating a probable association with drug-induced liver injury (Table 2 ).

**Table 2:** RUCAM score of the cases ALT: alanine transaminase, AST: aspartate transaminase, CT: computed tomography, CMV: cytomegalovirus, EBV: Epstein-Barr virus, HAV: hepatitis A virus, HBV: hepatitis B virus, HCV: hepatitis C virus, HEV: hepatitis E virus, MRC: magnetic resonance cholangiopancreatography, PCR: polymerase chain reaction, VZV: varicella zoster virus

Parameter	Case 1	Case 2	Case 3
1. Time to onset from the beginning of the drug/herb (5 to 90 days)	+2	+2	+2
2. Course of ALT after cessation of drug/herb (decrease ≥50% within 8 days)	+3	+3	+3
3. Risk factors	0	0	0
4. Concomitant drugs/herbs	0	0	0
5. Search for an alternative cause: Group 1 HAV, HBV, HCV, HEV, hepatobiliary sonography/color Doppler/endosonography/CT/MRC, alcoholism (AST/ALT ≥2), acute recent hypotension history. Group II complications of underlying diseases such as sepsis, metastatic malignancy, autoimmune hepatitis, primary biliary cholangitis, and genetic liver disease. Infection suggested by PCR and titer change for CMV, EBV, HSV, and VZV. All causes of groups 1 and II were reasonably ruled out.	+2	+2	+1
6. Previous hepatotoxicity of the drug	+1	+1	+1
7. Response to unintentional re-exposure	0	0	0
TOTAL	8	8	7

An online survey was conducted among gynecologists at our institute to assess their awareness of norethisterone-induced liver injury. A total of 15 gynecologists participated in the survey, and their experience ranged from 3 years to over 20 years. The results of this questionnaire are mentioned in Table 3. The most common indications for prescribing norethisterone were abnormal uterine bleeding and menorrhagia. One-third of the gynecologists use norethisterone as the first line, one-third use it as a second-line drug for menorrhagia, and one-third use it on case to case basis. Only 20% of them had come across any case of possible norethisterone-induced liver injury. Less than 50% of gynecologists order a baseline LFT before prescribing norethisterone. Around 30% monitor LFT at least once during follow-up. Around half of the gynecologists were not sure about the predominant type of liver injury induced by norethisterone.

**Table 3:** Results of the questionnaire LFT: liver function tests

Question	Response (%)		
Most common indication for norethisterone prescription?	Abnormal uterine bleeding (46.6%),		Menorrhagia (26.7%)
Status of norethisterone for menorrhagia?	First-line therapy (33.3%),	Second-line therapy (33.3%)	
Have you come across any case of possible norethisterone-induced liver injury?	No (80%)	Yes (20%)	
Do you order baseline liver function tests (LFT) before prescribing norethisterone?	Yes (46.7%)	No (53.3%)	
Do you monitor LFT in patients on norethisterone?	No (66.7%)	Yes (33.3%)	
What is the predominant form of norethisterone-induced liver injury?	Cholestatic (46.7%)	Not sure (53.3%)	

## Conclusions

Norethisterone-induced liver injury is a possibility and as demonstrated in our case series it should be a routine to do a liver function test both before and after initiating a female on this drug. Moreover, it is very important to create awareness about norethisterone-induced liver injury among clinicians, especially gynecologists. All patients, especially those with risk factors for drug-induced liver injury, like alcohol use or non-alcoholic fatty liver disease, should be more frequently monitored for liver dysfunction once started on norethisterone.
